# Guidance on the clinical understanding and use of long‐acting injectable antipsychotics in Schizophrenia: Hong Kong Consensus Statements

**DOI:** 10.1111/cns.13374

**Published:** 2021-02-08

**Authors:** Michael Ming Cheuk Wong, Albert Kar Kin Chung, Timothy Ming Hong Yeung, David Tai Wai Wong, Che Kin Lee, Eric Lai, Gloria Fong Yeung Chan, Gregory Kai Lok Mak, Jessica Oi Yin Wong, Roger Man Kin Ng, Ki Yan Mak

**Affiliations:** ^1^ Department of Psychiatry Queen Mary Hospital Pokfulam Hong Kong; ^2^ Private Practice Hong Kong City Hong Kong; ^3^ Private Practice Hong Kong City Hong Kong; ^4^ Department of Psychiatry Kowloon Hospital Kowloon Hong Kong; ^5^ Department of Child and Adolescent Psychiatry Castle Peak Hospital Tuen Mun New Territories Hong Kong; ^6^ Private Practice Hong Kong City Hong Kong; ^7^ Department of Psychiatry Castle Peak Hospital Tuen Mun Hong Kong; ^8^ The Mental Health Association of Hong Kong Private Practice Hong Kong

**Keywords:** consensus statements, guidance, long‐acting injectable antipsychotics, schizophrenia

## Abstract

**Aims:**

There is increasing evidence showing the importance of long‐acting injectable antipsychotics in the management of schizophrenia, especially in terms of improving patient medication compliance. A panel of experienced clinicians in Hong Kong mapped out a set of consensus statements with an aim to facilitate the understanding and use of long‐acting injectable antipsychotics among local physicians.

**Methods:**

Eight discussion areas regarding long‐acting injectable antipsychotics were selected by the chairman of the consensus group. A series of meetings were held for the panelists to discuss the published literature and their clinical experience, followed by the drafting of consensus statements. At the final meeting, each consensus statement was voted on anonymously by all members based on its practicability of recommendation in Hong Kong.

**Results:**

A total of 12 consensus statements on the rational use of long‐acting injectable antipsychotics were established and accepted by the consensus group.

**Conclusion:**

The consensus statements aim to provide practical guidance for Hong Kong physicians on the use of long‐acting injectable antipsychotics in schizophrenia patients. These statements may also serve as a reference for doctors in other parts of the Asia–Pacific region.

## 1 INTRODUCTION

Long‐acting injectable antipsychotics (LAIs) are important to improve the management of schizophrenia. A recent consensus statement by the Hong Kong Association of Psychosocial Rehabilitation indicates that there is growing concern about the need to tackle adherence issues in schizophrenia patients.^1^ An Asia‐Pacific survey revealed that 41% of Hong Kong psychiatrists prefer to switch or add a long‐lasting antipsychotic to address the adherence problem in their patients.^2^ In addition, there is evidence showing that cultural beliefs about mental illness and its treatment are an area for consideration in Hong Kong.^3^ This article aims to present the consensus statements established by a local expert panel, which is expected to facilitate the understanding and use of LAIs among Hong Kong physicians. Furthermore, these statements can hopefully act as a reference for doctors treating patients with schizophrenia in other parts of the Asia‐Pacific region.

## 2 METHOD

A consensus group of 11 Hong Kong physicians experienced in the management of schizophrenia were convened to formulate consensus statements on the rational use of LAIs, based on their clinical experience and the published literature. Eight discussion areas of LAIs were identified by the chairman of the consensus group, as follows: (a) characteristics; (b) special features; (c) comparison between first‐ and second‐generation antipsychotic LAIs; (d) efficacy; (e) special clinical uses; (f) tolerability and safety; (g) contraindications; and (h) patient education. A literature search was done on the PubMed database with the following keywords: ‘first‐generation antipsychotics’, ‘long‐acting injectable antipsychotics’, ‘paliperidone’, ‘risperidone’, ‘schizophrenia’, and ‘second‐generation antipsychotics’. Only articles published between 1996 and 2015 were included.

**Figure 1:**
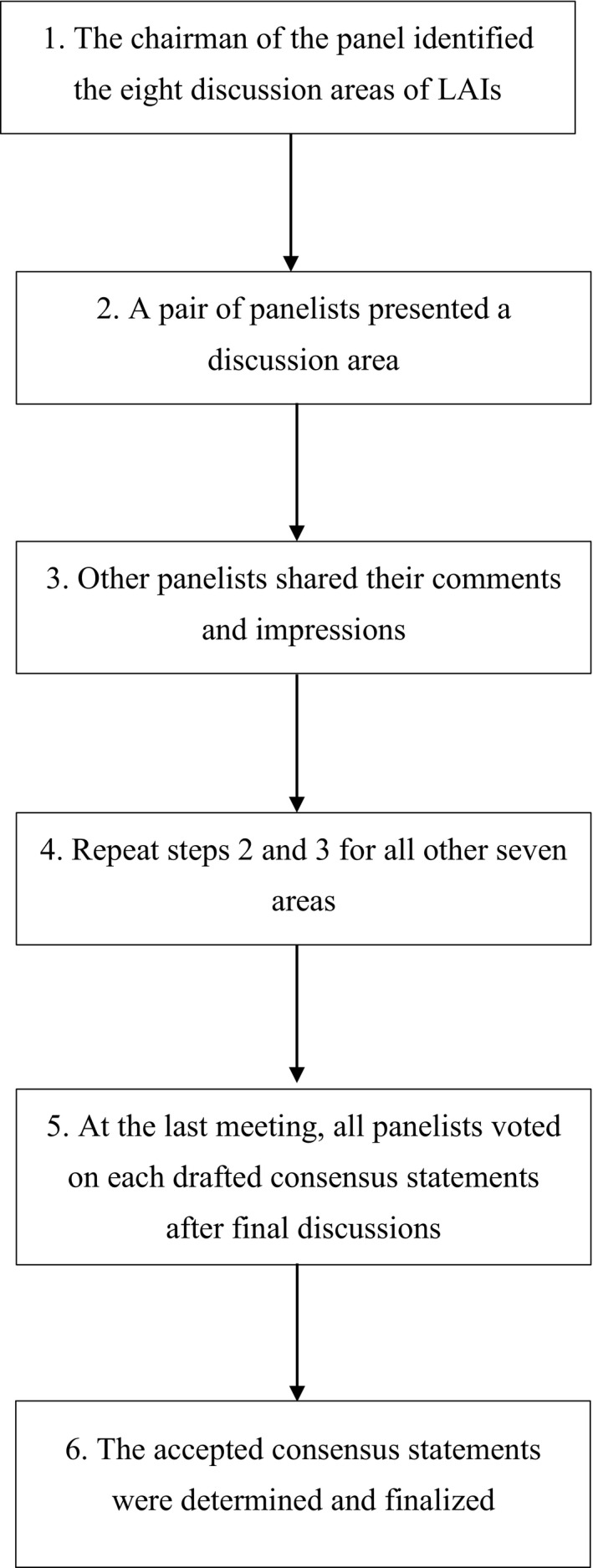
Process of consensus meetings

The consensus group utilized the modified Delphi technique^4^, ^5^ to hold a series of expert focus meetings (Figure 1). Each of the above discussion areas was presented by two panel members, including their clinical experience and the relevant literature, followed by comments contributed by other members. After all the areas were discussed, consensus statements were drafted based on the panelists’ comments and the published evidence. At the final meeting, each consensus statement was voted on anonymously by all members. With a method adapted from Ooi et al,^6^ each statement was assessed as per its practicability of recommendation in Hong Kong. A consensus statement was accepted only if ≥80% of the members selected “accept completely” (option A) or “accept with some reservation” (option B) for practicability (Table 1).

**Table 1:** Grading system for consensus statements^6^

Quality of evidence	Classification of recommendation	Practicability of recommendation
I: Evidence obtained from at least one randomized controlled trial	A: There is good evidence to support the statement	A: Accept completely
II‐1: Evidence obtained from well‐designed control trials without randomization	B: There is fair evidence to support the statement	B: Accept with some reservation
II‐2: Evidence obtained from well‐designed cohort or case‐control study	C: There is poor evidence to support the statement but recommendation made on other grounds	C: Accept with major reservation
II‐3: Evidence obtained from comparison between time or places with or without intervention	D: There is fair evidence to refute the statement	D: Reject with reservation
III: Opinion of respected authorities, based on clinical experience and expert committee	E: There is good evidence to refute the statement	E: Reject completely

Note

Modified from the Canadian Task Force on the Periodic Health Examination.

## 3 RESULTS

A total of 12 consensus statements regarding the rational use of LAIs were established and accepted by the consensus group.

Statement 1: The currently available second‐generation antipsychotic (SGA) LAIs in Hong Kong, paliperidone LAI (PLAI) and risperidone LAI (RLAI), differ in their formulations. An initial loading dose regimen of PLAI is required to achieve a rapid onset of actions and shorter time to achieve steady‐state levels. For RLAI, no loading dose is necessary, but at least 3 weeks of oral supplementations are required.

Voting results: A: 63%, B: 37%, C: 0%, D: 0%, E: 0%

Although aripiprazole LAI was recently approved for use in Hong Kong, it was not discussed by the consensus group because of its unavailability during the meeting timeframe. Olanzapine LAI is currently not commercially available in the locality. Therefore, the SGA LAIs mentioned in this article only included PLAI and RLAI, both of which had been available locally before the consensus group was established.

Paliperidone LAI is prepared as an aqueous suspension of nanocrystals of paliperidone palmitate.^7^, ^8^ With an increased surface area, the nanomolecules allow for rapid release, which shortens the time to a steady plasma level of active paliperidone after intramuscular injection.^7^ This kinetic property can also facilitate an initial loading regimen to reach therapeutic antipsychotic levels in schizophrenia patients, without the need for oral antipsychotics (oral APs).^8^ As per the results of some placebo‐controlled trials,^9^ starting or loading doses (ie, 150 mg equivalents [mg eq.] on day 1 and 100 mg eq. on day 8) are recommended to be administered in the deltoid muscle. Maintenance doses (ie, 25‐150 mg eq/4 wk) can be administered in the deltoid or gluteal muscle.

As for RLAI, it is a water‐based formulation of risperidone‐impregnated microspheres, which make loading impossible, and a lag period of about 3 weeks is observed during the drug‐releasing process.^7^, ^8^ Therefore, at least 3 weeks of supplemental oral APs should be prescribed initially to maintain a sufficient drug level.^7^, ^8^

Statement 2: Compared with first‐generation antipsychotic (FGA) LAIs, SGA LAIs are associated with a lower incidence of tardive dyskinesia and extrapyramidal syndrome; however, clinicians should beware of the increased risk for metabolic adverse events and weight gain.

Voting results: A: 50%, B: 50%, C: 0%, D: 0%, E: 0%

According to a number of reviews,^10^, ^11^, ^12^ the incidence of movement disorders, including tardive dyskinesia and extrapyramidal syndrome, is lower in patients receiving SGA LAIs, compared with those treated with FGA LAIs. This finding is generally consistent with the clinical experience of the consensus members.

However, some studies have shown that oral SGAs are associated with an increased risk of metabolic side effects and weight gain.^12^ These kinds of adverse events and other long‐term undesirable effects should be further investigated in patients receiving SGA LAIs.^11^ Physicians should be aware of the risk of metabolic events and weight gain in patients receiving SGA LAIs.

Statement 3: In terms of cognitive performance, SGA LAIs are preferred over oral antipsychotics and FGA LAIs. SGA LAIs are suggested to preserve white matter brain volume and provide a greater degree of neuroprotection.

Voting results: A: 63%, B: 37%, C: 0%, D: 0%, E: 0%

Two studies found that, compared with oral risperidone, RLAI may enhance the development of intracortical myelin and improve the myelination trajectory in first‐episode schizophrenia patients, possibly leading to a better cognitive function.^13^, ^14^ An open‐label trial revealed that a switch from haloperidol decanoate to RLAI may boost cognitive performance in patients.^15^ A review also found that SGA LAIs can be administered at an early stage of schizophrenia to better preserve cognitive capabilities of patients.^16^

In an in vitro study,^17^ risperidone and paliperidone were shown to possibly protect against apoptosis, while haloperidol induces it. The difference may be associated with their capacity to induce extrapyramidal symptoms. However, more studies are required to confirm the neuroprotective effect of SGA LAIs.

Statement 4: Contraindications to SGA LAIs are dependent on an individual patient's risk factors for the pharmacokinetics of the specific drug. PLAI and RLAI should be avoided in patients with severe renal or hepatic impairment.

Voting results: A: 37%, B: 63%, C: 0%, D: 0%, E: 0%

SGA LAIs are contraindicated in patients who are hypersensitive to the products and in the elderly with dementia‐associated psychosis.^18^, ^19^ Doctors should beware of other risk factors of individual patients, such as impaired renal or hepatic function, before prescribing an SGA LAI. It should be noted that PLAI is not recommended in patients having moderate or severe renal impairment (ie, creatinine clearance [CrCL] <50 mL/min),^20^ while RLAI should not be used in patients with severe renal impairment (ie, CrCL < 30 mL/min).^18^ Owing to the lack of clinical evidence, neither PLAI nor RLAI is recommended in patients suffering from severe hepatic impairment.^18^, ^20^, ^21^

Statement 5: SGA LAIs are not recommended in patients who are unresponsive and/or unable to tolerate the oral formulations. Cautions should be exercised in special patient groups such as children, pregnant women, the elderly, and patients with physical comorbidities.

Voting results: A: 100%, B: 0%, C: 0%, D: 0%, E: 0%

Before receiving SGA LAIs, patients should establish tolerability with the corresponding oral formulations, that is, risperidone or paliperidone.^18^, ^19^ It is not recommended to initiate SGA LAIs in patients who are unresponsive and/or intolerant to oral risperidone or paliperidone. In view of the absence of well‐received clinical evidence, the safety issues of SGA LAIs regarding special populations, which include pediatric patients, pregnant women, the elderly, and patients with physical comorbidities, may be a concern.^18^, ^19^, ^22^ A benefit‐risk analysis should be cautiously considered prior to the initiation of SGA LAIs in those special patient groups. More importantly, SGA LAIs are contraindicated in elderly patients who suffer from dementia‐related psychosis.^20^, ^21^ For other elderly patients, dose adjustment of SGA LAIs may be needed depending on their renal function.^20^, ^21^

Statement 6: Regardless of illness stage, SGA LAIs should be considered in all patients with schizophrenia as a shared decision‐making process in view of the vast evidence supporting the benefits on treatment outcome.

Voting results: A: 75%, B: 25%, C: 0%, D: 0%, E: 0%

As suggested in a number of reviews,^16^, ^23^, ^24^, ^25^ LAIs should be considered in all schizophrenia patients through a shared decision‐making process, irrespective of their stage of disease or adherence issues. Compared with oral APs, LAIs are found to be more beneficial to patients in terms of the more favorable safety profiles and significant improvements in patients’ psychotic symptoms, quality of life, and functioning.^26^, ^27^

In particular, several randomized controlled trials showed that, in comparison with oral APs, RLAI was associated with reductions in side effects, relapse, and hospitalization, as well as better adherence and enhanced clinical improvement.^28^, ^29^, ^30^ Some large‐scale observational studies also revealed the advantages of RLAI over other treatment options. A French study found that, after a 12‐month follow‐up, schizophrenia patients treated with RLAI had a 34% reduction in the hospitalization rate compared with those receiving other treatments, including FGA LAIs.^31^ In a 24‐month prospective study,^32^ compared with oral APs, RLAI was found to significantly improve treatment retention (81.8% for RLAI vs 63.4% for oral APs) and Clinical Global Impression Severity scores (−1.14 for RLAI vs −0.94 for oral APs) in patients with schizophrenia. In addition, a nationwide cohort study conducted in Finland showed that, among patients discharged from their first hospitalization for schizophrenia, the all‐cause discontinuation was significantly lower in those receiving RLAI compared with those treated with oral risperidone (hazard ratio = 0.44, 95% confidence interval 0.31‐0.62).^33^ It is noted that most of the current studies were conducted on RLAI, rather than PLAI, as the latter has only been approved for use for a relatively short period of time. Although it might be expected that PLAI and RLAI have comparable clinical benefits, further specific studies on PLAI are warranted.

Statement 7: The use of SGA LAls to enhance treatment adherence may be a reasonable choice at the early phase of treatment for first‐episode schizophrenic patients.

Voting results: A: 75%, B: 25%, C: 0%, D: 0%, E: 0%

The treatment adherence of first‐episode schizophrenia patients may be affected by their sensitivity to medication‐related adverse effects and inadequate insight about, or acceptance of, their illness.^34^, ^35^ Thus, the early stage of treatment may be pivotal in determining their disease progression in terms of clinical and psychosocial symptoms.^36^, ^37^

The results of several studies have suggested that SGA LAIs (primarily RLAI as it was the first to be marketed) are beneficial for first‐episode schizophrenia patients. In an open‐label study on a group of first‐episode patients treated with RLAI, 72% of them completed the trial, showing a relatively low discontinuation rate.^38^, ^39^ Moreover, 84% of them achieved a reduction of at least 50% on the Positive and Negative Syndrome Scale (PANSS) score, while 64% acquired a remission status as per the Remission in Schizophrenia Working Group (RSWG) remission criteria.^39^ Another open‐label study showed that, compared with oral risperidone, RLAI significantly reduced the relapse rate and enhanced medication adherence in first‐episode patients.^40^ Compared with other oral APs, RLAI was also found to be associated with improved acceptance and adherence in first‐episode patients.^34^

Based on the above clinical evidence and other research reviews,^41^, ^42^, ^43^, ^44^ the consensus group agreed that SGA LAIs may be a reasonable option for first‐episode schizophrenia patients in the early treatment stage, in order to improve medication adherence and other clinical outcomes.

Statement 8: The role of LAls should be considered for patients in the acute phase or with demonstrated signs of relapse, particularly in those with serious mental illness, lack of insight, lack of supportive carers, or for whom there are doubts about medication adherence.

Voting results: A: 88%, B: 12%, C: 0%, D: 0%, E: 0%

According to several major overseas guidelines and expert consensuses,^23^, ^25^, ^45^, ^46^, ^47^, ^48^ LAIs should be considered in schizophrenia patients with relatively severe conditions, such as those with signs of relapse, lack of insight, deficient family/social support, or adherence issues. The consensus group thereby recommend that local physicians should consider the use of LAIs instead of oral APs among such patients, so as to improve symptom control, relapse risk, and medication adherence.

Statement 9: For outpatients, in addition to those listed for the inpatients, LAIs are recommended to be used for patients that demonstrate signs of relapse and for whom there are doubts about medication adherence.

Voting results: A: 63%, B: 37%, C: 0%, D: 0%, E: 0%

As shown in the Finnish cohort study on patients discharged from hospitalization due to schizophrenia,^33^ those treated with LAIs had a significantly lower risk of re‐hospitalization compared with those treated with oral APs. An observational study also found that RLAI was correlated with reductions in both the number and days of hospitalization in schizophrenia patients compared with oral risperidone.^32^ The guidelines set by a group of French experts on psychiatry recommend SGA LAIs as first‐line treatment for patients receiving outpatient care.^25^ Based on the available evidence, the consensus group recommend that LAIs should be prescribed for patients in the outpatient setting who have signs of relapse and potential adherence issues, so as to reduce their hospitalization risk.

Statement 10: Because substance misuse predicts nonadherence to oral medications, the use of LAIs is recommended to enhance treatment adherence in schizophrenia patients with comorbid substance misuse.

Voting results: A: 100%, B: 0%, C: 0%, D: 0%, E: 0%

Substance misuse was found to be closely related to nonadherence to oral APs in patients with schizophrenia.^49^, ^50^ A set of clinical guideline recommendations derived from a literature review propose that LAIs are able to ensure treatment adherence in patients with comorbid substance misuse.^46^ The guideline from the National Institute for Health and Care Excellence (NICE) in the UK also recommends the use of LAIs in managing nonadherence problems among schizophrenia patients with coexisting substance misuse.^51^ The consensus group therefore agreed that, for the sake of medication adherence, LAIs are preferable to oral APs for schizophrenia patients with comorbid substance misuse.

Statement 11: The attitudes of the patients and their relatives toward LAIs should be addressed, and misconceptions are to be clarified via education, as part of the decision‐making process, tailored to the individual patient's need.

Voting results: A: 75%, B: 25%, C: 0%, D: 0%, E: 0%

According to a cross‐sectional study,^52^ patients currently on oral APs had a significantly lower preference for LAIs than those currently on LAIs. The researchers noted that possible patients’ concerns over the perceived forceful nature of LAI initiation should be addressed to tackle LAI underutilization. Other reasons why patients are negative toward LAIs may include the fear of autonomy restriction and the pain of injections.^53^ Some patients may also confuse LAIs with a short‐acting emergency intramuscular dose, resulting in an undesirable perception of LAIs.^54^ To increase patient acceptance of LAIs and diminish the negative image and stigma related to LAIs, clinicians should educate and inform patients and relatives sufficiently about this treatment option as part of a shared decision‐making process.^53^, ^54^, ^55^ Even if patients refuse LAIs initially, further discussion on LAIs at subsequent appointments could help them better understand the benefits.^46^, ^54^

Statement 12: Preclinical studies have shown the neurotoxicity of FGAs (especially haloperidol) and the neuroprotective effect of SGAs; clinicians should consider these when prescribing LAIs.

Voting results: A: 100%, B: 0%, C: 0%, D: 0%, E: 0%

Numerous preclinical studies on animal and human tissues showed that FGAs, in particular haloperidol, may cause neuron apoptosis and brain tissue destruction by reducing the number of cells in the prefrontal cortex, suggesting the presence of potential neurotoxicity.^17^, ^56^, ^57^ In contrast, SGAs, including olanzapine, risperidone, and paliperidone, were found to possibly enhance cell survival and neurogenesis in the prefrontal cortex, leading to a neuroprotective effect of apoptosis prevention.^17^, ^57^, ^58^, ^59^ In view of the current evidence, psychiatrists should beware of the possible difference in the mechanisms of action on brain tissues between FGAs and SGAs.

## 4 DISCUSSION

Long‐acting injectable antipsychotics, particularly SGA LAIs, have been found to be more beneficial than oral APs in multiple aspects of treatment of schizophrenia patients, including clinical symptom control, enhancement of treatment adherence, and reduction of relapse rate and hospitalization risk. LAIs are also applicable for patients in different stages of illness. In view of the importance of LAIs in the management of schizophrenia, the consensus group, through the discussion of the available literature and the panelists’ clinical experiences, established the above consensus statements, aiming to facilitate the rational use of LAIs among Hong Kong physicians.

Several limitations of these consensus statements have been noted. First, aripiprazole LAI, the latest approved SGA LAI in Hong Kong, was not discussed because it was not yet approved during the establishment of the consensus group. Its use in the locality should be further investigated by other local experts. Second, from the literature review, the clinical data on PLAI were significantly fewer than those on RLAI because of its rather short period of time on the market. More evidence regarding PLAI, especially from the local setting, is warranted to better assess its usage and properties. Third, local clinical trials on the use of LAIs are relatively scarce. Additional local data are required to further explore the pros and cons of LAIs among different types of schizophrenia patients in Hong Kong.

In conclusion, the consensus statements were formulated based on the available evidence from individual studies, expert consensuses, and major overseas guidelines, combined with the insights of the panelists. These statements aim to provide a practical guidance for local physicians on the use of LAIs in patients with schizophrenia.

## CONFLICT OF INTERESTS

The authors declare no conflict of interest.

## References

[1] Mak KY , Lo WT , Yeung WS , et al. Consensus statements on adherence issues in schizophrenia for Hong Kong. Asian J Psychiatr. 2014;12:163‐169. 25440570 10.1016/j.ajp.2014.06.018

[2] Olivares JM , Thirunavukarasu M , Kulkarni J , Zhang HY , Zhang M , Zhang F . Psychiatrists' awareness of partial and nonadherence to antipsychotic medication in schizophrenia: results from an Asia‐Pacific survey. Neuropsychiatr Dis Treat. 2013;9:1163‐1170. 23976858 10.2147/NDT.S49080 PMC3747021

[3] Bressington D , Mui J , Wells H . The effects of medication‐management training on clinicians' understanding and clinical practice in Hong Kong. Nurse Educ Today. 2013;33:969‐975. 23182892 10.1016/j.nedt.2012.10.021

[4] Leung WK , Ng SC , Chow DK , et al. Use of biologics for inflammatory bowel disease in Hong Kong: consensus statement. Hong Kong Med J. 2013;19:61‐68. 23378357

[5] Linstone HA , Turoff M . The Delphi Method: Techniques and Applications. Boston, MA: Addison‐Wesley Publishing Co., Inc; 2002.

[6] Ooi CJ , Fock KM , Makharia GK , et al. The Asia‐Pacific consensus on ulcerative colitis. J Gastroenterol Hepatol. 2010;25:453‐468. 20370724 10.1111/j.1440-1746.2010.06241.x

[7] Park EJ , Amatya S , Kim MS , et al. Long‐acting injectable formulations of antipsychotic drugs for the treatment of schizophrenia. Arch Pharm Res. 2013;36:651‐659. 23543652 10.1007/s12272-013-0105-7

[8] Meyer JM . Understanding depot antipsychotics: an illustrated guide to kinetics. CNS Spectr. 2013;18(Suppl 1):55–68. 10.1017/S1092852913000783 24345710

[9] Carter NJ . Extended‐release intramuscular paliperidone palmitate: a review of its use in the treatment of schizophrenia. Drugs. 2012;72:1137‐1160. 22571444 10.2165/11208640-000000000-00000

[10] Möller HJ . Long‐acting injectable risperidone for the treatment of schizophrenia: clinical perspectives. Drugs. 2007;67:1541‐1566. 17661527 10.2165/00003495-200767110-00003

[11] Bhanji NH , Chouinard G , Margolese HC . A review of compliance, depot intramuscular antipsychotics and the new long‐acting injectable atypical antipsychotic risperidone in schizophrenia. Eur Neuropsychopharmacol. 2004;14:87‐92. 15013023 10.1016/S0924-977X(03)00109-3

[12] Gopal S , Berwaerts J , Nuamah I , et al. Number needed to treat and number needed to harm with paliperidone palmitate relative to long‐acting haloperidol, bromperidol, and fluphenazine decanoate for treatment of patients with schizophrenia. Neuropsychiatr Dis Treat. 2011;7:93‐101. 21552311 10.2147/NDT.S17177 PMC3083982

[13] Bartzokis G , Lu PH , Amar CP , et al. Long acting injection versus oral risperidone in first‐episode schizophrenia: differential impact on white matter myelination trajectory. Schizophr Res. 2011;132:35‐41. 21767934 10.1016/j.schres.2011.06.029 PMC3172389

[14] Bartzokis G , Lu PH , Raven EP , et al. Impact on intracortical myelination trajectory of long acting injection versus oral risperidone in first‐episode schizophrenia. Schizophr Res. 2012;140:122‐128. 22809684 10.1016/j.schres.2012.06.036 PMC3567927

[15] Suzuki H , Gen K . The influence of switching from haloperidol decanoate depot to risperidone long‐acting injection on the clinical symptoms and cognitive function in schizophrenia. Hum Psychopharmacol. 2012;27:470‐475. 23001955 10.1002/hup.2249

[16] Brissos S , Veguilla MR , Taylor D , Balanzá‐Martinez V . The role of long‐acting injectable antipsychotics in schizophrenia: a critical appraisal. Ther Adv Psychopharmacol. 2014;4:198‐219. 25360245 10.1177/2045125314540297 PMC4212490

[17] Gassó P , Mas S , Molina O , Bernardo M , Lafuente A , Parellada E . Neurotoxic/neuroprotective activity of haloperidol, risperidone and paliperidone in neuroblastoma cells. Prog Neuropsychopharmacol Biol Psychiatry. 2012;36:71‐77. 21878360 10.1016/j.pnpbp.2011.08.010

[18] Janssen Pharmaceuticals Inc . RISPERDAL® CONSTA® Prescribing Information. Hong Kong version.

[19] Janssen Pharmaceuticals Inc . INVEGA SUSTENNA® Prescribing Information. Hong Kong version.

[20] Samtani MN , Gopal S , Gassmann‐Mayer C , Alphs L , Palumbo JM . Dosing and switching strategies for paliperidone palmitate: based on population pharmacokinetic modelling and clinical trial data. CNS Drugs. 2011;25:829‐845. 21936586 10.2165/11591690-000000000-00000

[21] Gopal S , Gassmann‐Mayer C , Palumbo J , Samtani MN , Shiwach R , Alphs L . Practical guidance for dosing and switching paliperidone palmitate treatment in patients with schizophrenia. Curr Med Res Opin. 2010;26;377‐387. 20001492 10.1185/03007990903482772

[22] Kim SW , Kim KM , Kim JM , et al. Use of long‐acting injectable risperidone before and throughout pregnancy in schizophrenia. Prog Neuropsychopharmacol Biol Psychiatry. 2007;31:543‐545. 17110011 10.1016/j.pnpbp.2006.09.017

[23] Moore TA , Buchanan RW , Buckley PF , et al. The Texas Medication Algorithm Project antipsychotic algorithm for schizophrenia: 2006 update. J Clin Psychiatry. 2007;68:1751‐1762. 18052569 10.4088/jcp.v68n1115

[24] Altamura AC , Aguglia E , Bassi M , et al. Rethinking the role of long‐acting atypical antipsychotics in the community setting. Int Clin Psychopharmacol. 2012;27:336‐349. 22859065 10.1097/YIC.0b013e328357727a

[25] Llorca PM , Abbar M , Courtet P , Guillaume S , Lancrenon S , Samalin L . Guidelines for the use and management of long‐acting injectable antipsychotics in serious mental illness. BMC Psychiatry. 2013;13:340. 24359031 10.1186/1471-244X-13-340 PMC3898013

[26] Taylor D . Psychopharmacology and adverse effects of antipsychotic long‐acting injections: a review. Br J Psychiatry Suppl. 2009;52:S13‐S19. 19880912 10.1192/bjp.195.52.s13

[27] Kaplan G , Casoy J , Zummo J . Impact of long‐acting injectable antipsychotics on medication adherence and clinical, functional, and economic outcomes of schizophrenia. Patient Prefer Adherence. 2013;7:1171‐1180. 24265549 10.2147/PPA.S53795 PMC3833623

[28] Chue P , Eerdekens M , Augustyns I , et al. Comparative efficacy and safety of long‐acting risperidone and risperidone oral tablets. Eur Neuropsychopharmacol. 2005;15:111‐117. 15572280 10.1016/j.euroneuro.2004.07.003

[29] Bai YM , Ting Chen T , Chen JY , et al. Equivalent switching dose from oral risperidone to risperidone long‐acting injection: a 48‐week randomized, prospective, single‐blind pharmacokinetic study. J Clin Psychiatry. 2007;68:1218‐1225. 17854246 10.4088/jcp.v68n0808

[30] Olivares JM , Pinal B , Cinos C . Comparison of long‐acting antipsychotic injection and oral antipsychotics in schizophrenia. Neuropsychiatry. 2011;1:275‐289.

[31] Grimaldi‐Bensouda L , Rouillon F , Astruc B , et al. Does long‐acting injectable risperidone make a difference to the real‐life treatment of schizophrenia? Results of the Cohort for the General study of Schizophrenia (CGS). Schizophr Res. 2012;134:187‐194. 22130111 10.1016/j.schres.2011.10.022

[32] Olivares JM , Rodriguez‐Morales A , Diels J , et al. Long‐term outcomes in patients with schizophrenia treated with risperidone long‐acting injection or oral antipsychotics in Spain: results from the electronic Schizophrenia Treatment Adherence Registry (e‐STAR). Eur Psychiatry. 2009;24:287‐296. 19195847 10.1016/j.eurpsy.2008.12.002

[33] Tiihonen J , Haukka J , Taylor M , Haddad PM , Patel MX , Korhonen P . A nationwide cohort study of oral and depot antipsychotics after first hospitalization for schizophrenia. Am J Psychiatry. 2011;168:603‐609. 21362741 10.1176/appi.ajp.2011.10081224

[34] Weiden PJ , Schooler NR , Weedon JC , Elmouchtari A , Sunakawa A , Goldfinger SM . A randomized controlled trial of long‐acting injectable risperidone vs continuation on oral atypical antipsychotics for first‐episode schizophrenia patients: initial adherence outcome. J Clin Psychiatry. 2009;70:1397‐1406. 19906343 10.4088/JCP.09m05284yel

[35] Coldham EL , Addington J , Addington D . Medication adherence of individuals with a first episode of psychosis. Acta Psychiatr Scand. 2002;106:286‐290. 12225495 10.1034/j.1600-0447.2002.02437.x

[36] Birchwood M , Todd P , Jackson C . Early intervention in psychosis. The critical period hypothesis. Br J Psychiatry Suppl. 1998;172:53‐59. 9764127

[37] Lieberman J , Jody D , Geisler S , et al. Time course and biologic correlates of treatment response in first‐episode schizophrenia. Arch Gen Psychiatry. 1993;50:369‐376. 8098203 10.1001/archpsyc.1993.01820170047006

[38] Emsley R , Medori R , Koen L , Oosthuizen PP , Niehaus DJ , Rabinowitz J . Long‐acting injectable risperidone in the treatment of subjects with recent‐onset psychosis: a preliminary study. J Clin Psychopharmacol. 2008;28:210‐213. 18344732 10.1097/JCP.0b013e318167269d

[39] Emsley R , Oosthuizen P , Koen L , Niehaus DJ , Medori R , Rabinowitz J . Remission in patients with first‐episode schizophrenia receiving assured antipsychotic medication: a study with risperidone long‐acting injection. Int Clin Psychopharmacol. 2008;23:325‐331. 18854720 10.1097/YIC.0b013e32830c2042

[40] Kim B , Lee SH , Choi TK , et al. Effectiveness of risperidone long‐acting injection in first‐episode schizophrenia: in naturalistic setting. Prog Neuropsychopharmacol Biol Psychiatry. 2008;32:1231‐1235. 18442879 10.1016/j.pnpbp.2008.03.012

[41] Jeong HG , Lee MS . Long‐acting Injectable Antipsychotics in First‐episode Schizophrenia. Clin Psychopharmacol Neurosci. 2013;11:1‐6. 23678347 10.9758/cpn.2013.11.1.1 PMC3650291

[42] Taylor M , Ng KY . Should long‐acting (depot) antipsychotics be used in early schizophrenia? A systematic review. Aust N Z J Psychiatry. 2013;47:624‐630. 23209308 10.1177/0004867412470010

[43] Přikryl R , Přikrylová Kučerová H , Vrzalová M , Cešková E . Role of long‐acting injectable second‐generation antipsychotics in the treatment of first‐episode schizophrenia: a clinical perspective. Schizophr Res Treatment. 2012;2012:764769. 22966444 10.1155/2012/764769 PMC3420571

[44] Parellada E , Velligan DI , Emsley R , Kissling W . Long‐acting injectable antipsychotics in first‐episode schizophrenia. Schizophr Res Treatment. 2012;2012:318535. 22966433 10.1155/2012/318535 PMC3432356

[45] NICE . Schizophrenia: Core Interventions in the Treatment and Management of Schizophrenia in Primary and Secondary Care (update). NICE Clinical Guidelines No. 82. London, UK: National Institute for Health and Care Excellence,2009.

[46] Kane JM , Garcia‐Ribera C . Clinical guideline recommendations for antipsychotic long‐acting injections. Br J Psychiatry Suppl. 2009;52:S63‐S67. 19880920 10.1192/bjp.195.52.s63

[47] Velligan DI , Weiden PJ , Sajatovic M , et al. Strategies for addressing adherence problems in patients with serious and persistent mental illness: recommendations from the expert consensus guidelines. J Psychiatr Pract. 2010;16:306‐324. 20859108 10.1097/01.pra.0000388626.98662.a0

[48] Koola MM , Wehring HJ , Kelly DL . The Potential Role of Long‐acting Injectable Antipsychotics in People with Schizophrenia and Comorbid Substance Use. J Dual Diagn. 2012;8:50‐61. 22754405 10.1080/15504263.2012.647345 PMC3383636

[49] Fenton WS , Blyler CR , Heinssen RK . Determinants of medication compliance in schizophrenia: empirical and clinical findings. Schizophr Bull. 1997;23:637‐651. 9366000 10.1093/schbul/23.4.637

[50] Lacro JP , Dunn LB , Dolder CR , Leckband SG , Jeste DV . Prevalence of and risk factors for medication nonadherence in patients with schizophrenia: a comprehensive review of recent literature. J Clin Psychiatry. 2002;63:892‐909. 12416599 10.4088/jcp.v63n1007

[51] NICE . Coexisting severe mental illness (psychosis) and substance misuse: assessment and management in healthcare settings. Clinical guideline [CG120], 2011 https://www.nice.org.uk/guidance/cg120. Accessed 25 May 2017. 31851442

[52] Patel MX , De Zoysa N , Bernadt M , David A . Depot and oral antipsychotics: patient preferences and attitudes are not the same thing. J Psychopharmacol. 2009;23:789‐796. 18583438 10.1177/0269881108092124

[53] Jaeger M , Rossler W . Attitudes towards long‐acting depot antipsychotics: a survey of patients, relatives and psychiatrists. Psychiatry Res. 2010;175:58‐62. 20004980 10.1016/j.psychres.2008.11.003

[54] Bera RB . Patient outcomes within schizophrenia treatment: a look at the role of long‐acting injectable antipsychotics. J Clin Psychiatry. 2014;75(Suppl 2):30‐33. 24919169 10.4088/JCP.13065su1c.07

[55] Kirschner M , Theodoridou A , Fusar‐Poli P , Kaiser S , Jäger M . Patients’ and clinicians’ attitude towards long‐acting depot antipsychotics in subjects with a first episode of psychosis. Ther Adv Psychopharmacol. 2013;3:89‐99. 24167680 10.1177/2045125312464106 PMC3805393

[56] Nasrallah HA . Haloperidol clearly is neurotoxic. Should it be banned? Curr Psychiatr. 2013;12:7‐8.

[57] Nandra KS , Agius M . The differences between typical and atypical antipsychotics: the effects on neurogenesis. Psychiatr Danub. 2012;24(Suppl 1):S95‐S99. 22945197

[58] Wang HD , Dunnavant FD , Jarman T , Deutch AY . Effects of antipsychotic drugs on neurogenesis in the forebrain of the adult rat. Neuropsychopharmacology. 2004;29:1230‐1238. 15085089 10.1038/sj.npp.1300449

[59] Wakade CG , Mahadik SP , Waller JL , Chiu FC . Atypical neuroleptics stimulate neurogenesis in adult rat brain. J Neurosci Res. 2002;69:72‐79. 12111817 10.1002/jnr.10281

